# Congenital heart defects – diagnosis and treatment

**DOI:** 10.1038/s41598-026-63957-3

**Published:** 2026-07-25

**Authors:** Robert S. Kass, Olivier Villemain, Lincai Ye

**Affiliations:** 1 Department of Molecular Pharmacology, Columbia University, New York, NY 10032 USA; 2 Department of Pediatric and Adult Congenital Cardiology, Bordeaux University Hospital (CHU), Pessac, France; 3 Institut Hospital-Universitaire Liryc, Fondation Bordeaux Université, Av. du Haut Lévêque, 33600 Pessac, France; 4 Shanghai Institute for Pediatric Congenital Heart Disease, Shanghai Children’s Medical Center, Shanghai Jiao Tong University School of Medicine, Shanghai, 200127 China; 5 Institute of Pediatric Translational Medicine, Shanghai Children’s Medical Center, School of Medicine, Shanghai Jiao Tong University, Shanghai, 200127 China

**Keywords:** Congenital heart defects, Cell biology

## Abstract

More than six decades ago, the first successful repair of a congenital heart defect opened a new frontier in pediatric medicine. Driven by advances in fetal imaging, computational fluid dynamics, machine learning, and surgical perfusion, our ability to diagnose and treat these conditions has grown remarkably. Many of today’s greatest challenges in congenital cardiology - early and accurate diagnosis, precise risk stratification, and lifelong management - are increasingly being addressed through methods borrowed from engineering and data science. The Congenital heart defects: Diagnosis and treatment Collection at Scientific Reports is dedicated to this research.

## Introduction

Congenital heart defects (CHDs) represent the most common category of birth defects, affecting nearly 1% of live births worldwide^1^. Over the past decades, remarkable strides have been made in prenatal screening^2^, noninvasive imaging^3^, surgical techniques^4^, and perioperative care^5^, transforming outcomes for children born with even the most complex cardiac anomalies^6^. Yet challenges remain: early and accurate diagnosis in low-resource settings, optimization of risk stratification, reduction of postoperative morbidity, and long-term management of growing populations of CHD survivors. This Collection, Congenital heart defects: Diagnosis and treatment, brings together a diverse set of primary research articles that address these pressing issues, spanning from fetal reference values to artificial intelligence‑driven auscultation and from hemodynamic modeling to innovative perfusion strategies.

The six papers published in this Collection illustrate the breadth and depth of current investigation in the field. Several studies focus on refining diagnostic tools -whether through automated murmur detection^7^, normative fetal data^8^, or computational fluid dynamics (CFD)^9^. Others tackle critical aspects of surgical and perioperative management, including mortality prediction models^10^, cytokine modulation during cardiopulmonary bypass^11^, and physiological assessment after high‑risk palliation^12^. Collectively, they highlight a trajectory toward more personalized, data‑driven, and less invasive care for patients with CHDs.

In this editorial, we will briefly introduce three particularly noteworthy contributions, each representing a distinct pillar of contemporary CHD research: diagnostic innovation, hemodynamic precision, and prognostic modeling. Finally, weoffer some perspectives on future directions that may further reshape the landscape.

Early detection of CHDs in pediatric populations often begins with cardiac auscultation -a skill that requires extensive training and is subject to inter‑observer variability. Hsieh and colleagues^7^ address this gap by developing and validating an integrated residual‑recurrent neural network model for automated heart murmur detection. Their model, trained on a large cohort of pediatric heart sounds, achieves high sensitivity and specificity, offering a scalable, low‑cost screening tool that could be deployed in primary care or even home‑based settings. The clinical implications are substantial: timely identification of pathological murmurs can expedite echocardiographic confirmation and surgical referral, reducing the risk of missed diagnoses in resource‑limited environments. Moreover, this work exemplifies how artificial intelligence can augment, rather than replace, clinical acumen -freeing specialists to focus on complex decision‑making while improving access to basic cardiac screening.

Coarctation of the aorta remains a common and potentially life‑threatening CHD, yet conventional imaging modalities (echocardiography, MRI, CT) provide only static anatomical or averaged flow information. Hu and co‑workers^9^ present a sophisticated approach that combines multimodal imaging with CFD to noninvasively assess hemodynamic severity. By reconstructing patient‑specific three‑dimensional geometries from MRI or CT and simulating pulsatile blood flow, they derive parameters such as pressure gradients, wall shear stress, and energy loss that correlate closely with invasive catheterization measurements. This technique not only refines the selection of candidates for intervention but also enables virtual planning of balloon angioplasty or stent placement. For patients with recurrent or borderline coarctation, serial CFD assessments may guide the timing of reintervention with unprecedented accuracy. The study is a compelling example of how computational modeling is moving from research laboratories into clinical decision support.

Risk stratification before congenital heart surgery is essential for counseling families, allocating resources, and designing quality improvement initiatives. An and colleagues^10^ developed and externally validated a prediction model for postoperative mortality using two large‑scale, multi‑institutional cohorts. Their model incorporates a parsimonious set of preoperative variables -including age, weight, diagnostic category, prior interventions, and markers of organ dysfunction -and demonstrates excellent discrimination and calibration across diverse populations. Unlike earlier risk scores that were derived from single‑center or outdated data, this work leverages contemporary cohorts and rigorous statistical methods, including handling of missing data and internal‑external cross‑validation. The resulting tool can be implemented as a bedside nomogram or web‑based calculator, providing real‑time risk estimates that may inform perioperative management and family discussions. This study represents a benchmark for evidence‑based surgical decision support in pediatric cardiac surgery.

While space precludes detailed discussion of every article, the remaining three studies deserve mention. Poertecene et al.^11^ demonstrated that pre‑bypass ultrafiltration of blood prime significantly reduces cytokine burden in pediatric cardiac surgery, potentially mitigating the systemic inflammatory response associated with cardiopulmonary bypass. Aronsson and colleagues^12^provided a refined method for determining cardiac output, shunt fraction, and active circulatory volume in infants with hypoplastic left heart syndrome after the Norwood procedure -critical parameters for managing these fragile patients in the intensive care unit. Finally, Phuakpoolpol et al^8^. established gestational age‑specific reference values for fetal pulmonary artery branch diameters from 18 to 40 weeks, filling an important gap in prenatal sonographic assessment.

## Concluding remarks

Looking ahead, several trends will likely shape the next decade of CHD care. First, the integration of artificial intelligence and machine learning will extend beyond murmur detection and risk prediction into real‑time image interpretation, automated echocardiographic measurement, and individualized treatment recommendation^13^. Second, computational modeling -as illustrated by Hu et al.^9^ -will become more accessible through cloud‑based platforms and reduced computation times, enabling virtual surgery planning and hemodynamic simulation as routine clinical tools^14^. Third, the growing recognition of neurodevelopmental and psychosocial outcomes in CHD survivors calls for a lifespan approach, linking early surgical data with long‑term follow‑up registries^15^. Fourth, advances in fetal intervention and minimally invasive hybrid procedures may further reduce the burden of open heart surgery in the youngest patients^16^. Finally, global health equity must remain a priority^17,18^: many of the diagnostic and therapeutic innovations showcased in this Collection remain unavailable in low‑ and middle‑income countries, where the majority of CHD births occur. Scalable, low‑cost solutions -such as AI‑enabled stethoscopes, task‑shifting in echocardiography, and simplified perfusion techniques -will be essential to close this gap.

The journey toward optimal outcomes for every child with a CHD is far from complete, but the path forward is increasingly illuminated by evidence, innovation, and collaboration.
